# Regulation of Melanogenesis in Skin Epidermal Keratinocytes via Activation of α7 Nicotinic Acetylcholine Receptor on the Expression of α‐Melanocyte‐Stimulating Hormone

**DOI:** 10.1096/fj.202502535R

**Published:** 2025-10-12

**Authors:** Maggie Suisui Guo, Xiaoyang Wang, Yingjie Xia, Jiahui Wu, Ajiaikebaier Dilidaer, Jin Gao, Tina Tingxia Dong, Karl Wah Keung Tsim

**Affiliations:** ^1^ Division of Life Science, Center for Chinese Medicine and State Key Laboratory of Molecular Neuroscience The Hong Kong University of Science and Technology Hong Kong China; ^2^ Shenzhen Key Laboratory of Edible and Medicinal Bioresources, SRI The Hong Kong University of Science and Technology Shenzhen China; ^3^ Department of Neurobiology and Cellular Biology Xuzhou Medical University Xuzhou China

**Keywords:** acetylcholine receptor, alpha‐MSH, cholinergic effects, melanogenesis, POMC

## Abstract

UV irradiation stimulates the production of pro‐opiomelanocortin (POMC) and its derived peptides, for example, α‐melanocyte stimulating hormone (α‐MSH), from keratinocytes that subsequently stimulate melanin production in melanocytes. The idea of “skin synapse” describes the interplay between keratinocytes and melanocytes being modulated by cholinergic signaling, in which acetylcholine (ACh) release is induced by UV irradiation, regulating the process of pigmentation. ObjectiveHere, the role of the cholinergic system in regulating α‐MSH production in keratinocytes during UV‐induced skin pigmentation was identified. α7 Nicotinic acetylcholine receptor (nAChR) agonists and antagonists, Ca^2+^ chelator BAPTA‐AM, and α7 nAChR shRNA were applied onto HaCaT keratinocytes to evaluate the expression of POMC and production of α‐MSH following UVB induction. Moreover, the conditioned medium collected from the treated HaCaT keratinocytes was added to cultured B16F10 melanoma cells to assess melanogenesis. Inhibiting α7 nAChR by its antagonist, or Ca^2+^ influx by Ca^2+^ chelator, suppressed the UVB‐induced POMC expression and α‐MSH production in cultured keratinocytes. Genetic silencing α7 nAChR expression hindered the UVB‐induced POMC expression. Besides, the conditioned medium collected from keratinocytes being treated with α7 nAChR antagonist and UVB downregulated melanogenesis, as implied by the reduced expression of melanogenic enzymes, indicating the suppressing effect of α7 nAChR antagonist on the production of α‐MSH in keratinocytes. The result suggests that α7 nAChR mediates the regulation of melanogenesis through the modulation of UVB‐induced POMC transcription and α‐MSH production in keratinocytes.

## Graphical abstract

α7 nAChR modulates UVB‐induced α‐MSH production during melanogenesis. UVB irradiation induces DNA damage in epidermal keratinocytes, causing upregulation of POMC transcription via the increase of its transcription factor p53, which in turn leads to elevated α‐MSH mastering melanin synthesis in epidermal melanocytes. During this process, ACh released from keratinocytes works on α7 nAChR, triggering Ca^2+^ mobilization and participating in the expression of POMC. By antagonizing the α7 nAChR with MLA, the production of POMC and α‐MSH is suppressed. Consequently, melanogenesis in melanocytes is hindered.

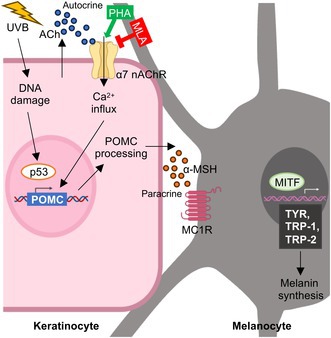

## 1 Introduction

Ultraviolet (UV) is a direct ambient cause of skin pigmentation, which triggers the synthesis of melanin in the skin epidermis. As the outermost layer of skin, epidermal keratinocytes act as a primary barrier against UV irradiation. UV radiation causes cell injury, as marked by an activation of p53 expression. The transcription factor p53 binds to the promoter region of pro‐opiomelanocortin (POMC) and initiates its transcription [1, 2, 3]. POMC is a multicomponent precursor of hormones and neuropeptides. Derived hormones include α‐melanocyte stimulating hormone (α‐MSH), adrenocorticotropic hormone (ACTH), and the opioid peptide β‐endorphin, etc. [1]. Among them, α‐MSH is the functional peptide that participates in the synthesis of melanin in epidermal melanocytes by acting on the melanocortin receptor on melanocytes. The synthesized melanin functions in preventing epidermal cells from further UV damage.

During melanogenesis, α‐MSH binds to melanocortin 1 receptor (MC1R) present on melanocytes. The activation of MC1R turns on the cyclic adenosine monophosphate (cAMP) signaling, followed by the transcription of microphthalmia‐associated transcription factor (MITF). Under this signaling cascade, MITF regulates the expression of melanogenic genes in melanocytes, including tyrosinase (TYR), tyrosine‐related protein‐1 (TRP‐1), and dopachrome tautomerase (DCT), or namely TRP‐2. These genes encode the enzymes that are mainly involved in the transformation of tyrosine to melanin in the organelle melanosomes [4, 5]. Following that, the fully melanized melanosomes are transferred from melanocytes to keratinocytes, subsequently aggregating at the perinucleus regions of keratinocytes and serving a role of photo‐protection [6, 7].

Cholinergic system utilizes the neurotransmitter acetylcholine (ACh) in transmitting signals between cells, in which the cholinergic synapse is formed [8]. Cells adopting the cholinergic system for signal transmission are found in different regions of the nervous system, regulating cognitive functions, as well as the activity of muscles [9]. Other than that, we reported the non‐neuronal roles of the cholinergic system in influencing skin pigmentation. In the proposed idea of “skin synapse,” UV irradiation elicits the release of ACh from epidermal cells, mediating the cellular events including melanosome release and uptake via certain ACh receptors [10, 11, 12]. In keratinocytes, α7 nicotinic acetylcholine receptor (nAChR) plays a role in coordinating the uptake of melanosomes through the control of cellular actin dynamics [10].

In the brain, the hypothalamic POMC neurons found in the arcuate nucleus were reported to have a cholinergic phenotype. Production of α‐MSH by POMC neurons can be regulated by hypothalamic ACh or injected nicotine, via nAChR activation [13, 14]. Here, we hypothesize that in epidermal keratinocytes, nAChR could have similar regulatory roles on the expressions of POMC and α‐MSH during UV induction. Translational and transcriptional levels of POMC and α‐MSH were assessed. Besides, the detailed mechanisms by which POMC expression is modulated by the cholinergic system were investigated in this study.

## 2 Materials and Methods

### 2.1 Materials

Dulbecco's modified Eagle medium (DMEM), fetal bovine serum (FBS), and reagents for cell culture were purchased from Thermo Fisher Scientific (Waltham, MA, USA). Antibodies were purchased from Abcam Ltd. (Cambridge, UK) and Cell Signaling Technology (CST; Danvers, MA, USA). RNAzol RT reagent and other chemicals were purchased from Sigma‐Aldrich (St. Louis, MO, USA). ELISA kits were purchased from Fankew (Shanghai, China).

### 2.2 Cell Culture

Human keratinocyte HaCaT and murine melanoma B16F10 cell lines, from ATCC (American Type Culture Collection; Manassas, VA, USA), were supplied with DMEM culture medium, supplemented with 10% FBS as well as 1% penicillin/streptomycin (P/S; 100 U/mL and 100 mg/mL). The cultures were kept in a CO_2_ incubator, in a condition of 37°C, 7.5% CO_2_, and saturated humidity. Cells were brought to sub‐culture at a confluency of around 80%.

### 2.3 Measurement of Cell Viability

The cell viability after treatments was tested using 3‐(4,5‐Dimethylthiazol‐2‐yl)‐2,5‐diphenyltetrazolium bromide (MTT). HaCaT keratinocytes were irradiated by UVB at different durations. Narrowband UVB lamp TL 20W/01 (Philips) was used for UVB irradiation. The cells were irradiated by UVB at the dose of 200 J/m^2^: the dose was measured with an Optometer and XD‐9509 detector (Gigahertz‐Optik GmbH, Germany). After 6 h of incubation, MTT in 0.5 mg/mL was added. After 3 h of MTT incubation, dimethyl sulfoxide (DMSO) was added to dissolve the purple formazan formed in the culture. The absorbance at 570 nm was measured with a microplate reader.

### 2.4 Quantitative Real‐Time PCR

RNAzol RT reagent (Molecular Research Center, Cincinnati, OH, USA) was used for RNA extraction. Following RNA extraction, reverse transcription on the samples was performed using commercial kit from Takara Bio (Shiga, Japan). In brief, 500 ng RNA, 5× reverse transcriptase master mix, and RNAase‐free water added to 10‐μL reaction volume were subjected to 37°C for 15 min. The reaction was terminated by putting onto 85°C for 5 s. As to quantify the relative expression level of target genes, real‐time quantitative polymerase chain reaction (qPCR) was performed in the Roche Lightcycler 480 system (Roche, Basel, Switzerland). The PCR temperature cycles were programmed as pre‐denaturation stage: 95°C, 30 s; denaturation: 95°C, 10 s; annealing temperature: 55°C, 30 s; elongation: 72°C, 30 s. There were in total 45 cycles. The specificity of amplification was confirmed by a melting curve. The transcript levels of each target gene were normalized with GAPDH by calculating 2^−△△Ct^ values. The PCR primers employed were as below: Human GAPDH Forward: 5′‐ACA ACT TTG GTA TCG TGG AAG G‐3′, Reverse: 5′‐GCC ATC ACG CCA CAG TTT C‐3′; POMC Forward: 5′‐GGC TTG CAA ACT CGA CCT CT‐3′, Reverse: 5′‐TGA CCC ATG ACG TAC TTC CG‐3′. The primers for melanogenic genes were as described, including Mouse GAPDH Forward: 5′‐AAG GTC ATC CCA GAG CTG AA‐3′, Reverse: 5′‐GCC ATG ACG CCA CAG TTT C‐3′; TYR Forward: 5′‐AGT CGT ATC TGG CCA TGG CTT CTT G‐3′, Reverse: 5′‐GCA AGC TGT GGT AGT CGT CTT TGT C‐3′; TRP‐1 Forward: 5′‐CTG CGA TGT CTG CAC TGA TGA CTT G‐3′, Reverse: 5′‐TTT CTC CTG ATT GGT CCA CCC TCA‐3′; TRP‐2 Forward: 5′‐TTG TCA CGT GGC ACA GGT ACC ATC‐3′, Reverse 5′‐CAG CGT TGG GTC ATC TTG TCT TGC‐3′; MITF Forward: 5′‐AGF AGA AGC TGG AGC ATG CGA ACC‐3′, Reverse 5′‐GTT CCT GGC TGC AGT TCT CAA GAA C‐3′ [15].

### 2.5 DNA Transfection

Transient transfection of DNA constructs on cultured cells was performed. Commercial kit jetPRIME Transfection kit (Polyplus, Lyon, France) was used. The protocol attached to the kit was followed. Transfected cells were harvested or subjected to drug treatments. The promoter constructs, pP53‐Luc and pPOMC‐Luc, were gifts from Wafik El‐Deiry (pGL2‐356bp; Addgene plasmid #16292) and Domenico Accili (Pomc‐pGL3; Addgene plasmid #17553), respectively [16, 17]. The α7 nAChR shRNA was purchased from Santa Cruz Biotechnology (Santa Cruz, CA, USA). The reporter constructs, pMITF‐Luc and pTYR‐Luc, were described in the previous work [15].

### 2.6 Luciferase Reporter Assay

The promoter activity of target genes was quantified using a Luciferase Assay System (Promega). Cells were transfected with a construct containing the target gene promoter fused to a luciferase reporter gene. The adopted constructs included pMITF‐Luc and pTYR‐Luc. In brief, the transfected cells, after treatment, were lysed and subjected to agitation at 4°C for 15 min. The lysates were centrifuged at 13 200 rpm for 5 min in microcentrifuge tubes. Supernatants were kept on ice and subjected to the luciferase reporter assay. The luminous signals were captured and quantified by GloMax 96 microplate luminometer (Promega, Madison, WI, USA). The protein concentration of each collected sample was determined by Bradford assay, and the luciferase measurements were normalized to the protein concentration for each corresponding sample.

### 2.7 Western Blotting Analysis

Cells after treatment were subjected to protein extraction and Bradford assay as described previously [11]. Each sample of 20‐μg protein was resolved by sodium dodecyl sulfate polyacrylamide gel electrophoresis (SDS‐PAGE) and transferred to nitrocellulose membranes for western blotting. The blots were blocked with 5% skim milk for 1 h at room temperature. Subsequently, they were subjected to overnight primary antibody incubation at 4°C, followed by 2 h secondary antibody incubation at room temperature. Primary antibodies against POMC, α7 nAChR, tyrosinase, TRP‐1, and α‐tubulin (DM1A), as well as secondary mouse and rabbit IgG horseradish peroxidase (HRP)‐linked antibodies were used.

### 2.8 Enzyme‐Linked Immunosorbent Assay (ELISA)

The amount of target protein released in culture medium was determined by ELISA. The collected culture medium was centrifuged at 1000*× g* for 15 min at 4°C in microcentrifuge tubes. Supernatant was analyzed by ELISA following the manufacturer's instructions. In brief, samples and protein standards were added to the antibody‐coated wells. After adding enzyme conjugate, the plate was incubated in a humidified chamber at 37°C for 1 h. After that, the wells were washed 5 times with 400 μL of wash buffer (15–30 s per wash). Fifty microliters of substrate A and B were added for color development. Stop solution was applied to each well following 15 min of incubation at 37°C in the dark. Absorbance at 450 nm was measured in a microplate reader. The target protein concentration was calculated from the standard curve.

### 2.9 Statistical Analysis

Mean ± standard deviation (SD) was adopted in expressing the data. Statistical tests were performed by one‐way ANOVA with the statistical analytic software GraphPad Prism 8. In each statistical analysis, differences from blank treatment values were classified as *p < 0.05; **p < 0.01; ***p < 0.001. Differences from UVB treatment values were classified as ^#^ *p* < 0.05; ^##^ *p* < 0.01; ^###^ *p* < 0.001. The number of independent biological replicates was presented by value *n*, which was described in the figure legends.

## 3 Result

### 3.1 UVB Induces POMC Expression in HaCaT Cells

**FIGURE 1:**
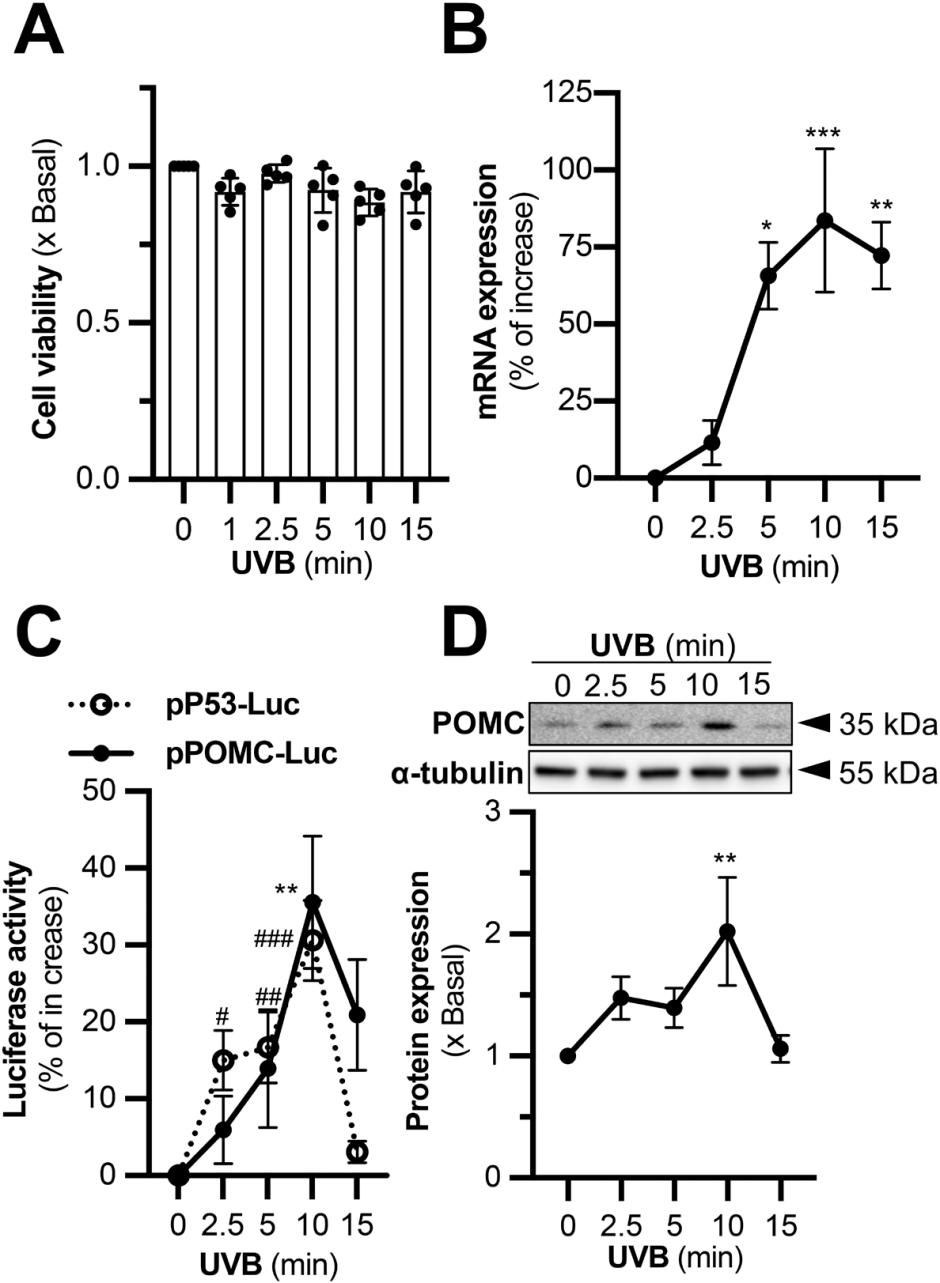
POMC expression is induced by UVB irradiation in HaCaT keratinocytes. UVB irradiation of different exposure durations, that is, 0, 2.5, 5, 10, 15 min, was applied to HaCaT keratinocytes. (A) Cells were seeded onto several 35‐mm dishes in a density of 1 × 10^5^ cells/mL. MTT assay was performed to assess the cell viability. (B) Cells were seeded onto 12‐well plates in a density of 1.5 × 10^5^ cells/mL. They were collected for RNA extraction, reverse transcription and subsequently subjected to RT‐qPCR to measure the relative expression of POMC to GAPDH as the internal control. (C) UVB treatment was applied to HaCaT cells transiently transfected with DNA construct pP53‐Luc or pPOMC‐Luc. The cell lysates were subjected to luciferase promoter assay. (D) Western blotting assay was performed to quantify the expressions of POMC (~35 kDa) and α‐tubulin (~55 kDa) proteins. Blotted band intensity was quantified and compared. Values are expressed as fold of change (*x* basal) or percentage of increase, in Mean ± SD, *n* = 5. Statistical significance was analyzed by one‐way ANOVA, *p < 0.05, **p < 0.01, ***p < 0.001; or ^#^ *p* < 0.05, ^##^ *p* < 0.01, ^###^ *p* < 0.001 versus the blank control.

A narrowband UVB lamp was adopted to induce POMC expression, using HaCaT keratinocytes as the cell model. MTT assay was first performed to study the cytotoxicity effect of UVB irradiation of different exposure durations (1–15 min). Observed at 6 h post‐irradiation, the viability of cells remained unchanged by all the doses tested, compared to the blank control (Figure 1A). The UVB exposure duration required for an effective induction of POMC expression was determined at the transcriptional and translational level. Real‐time qPCR results revealed a time‐dependent increase in POMC mRNA, with a peak observed following 10 min UVB exposure, which was over a 75% increase compared to control (Figure 1B). This transcriptional activation following 10 min irradiation was confirmed using the POMC promoter‐driven luciferase assay (Figure 1C). Notably, the promoter activity of p53, the known transcription factor of POMC, demonstrated a similar expression pattern following UVB irradiation. An obvious drop in the promoters' activities was observed following 15 min irradiation (Figure 1C). Western blotting analysis reflected a similar trend, showing approximately a 2‐fold increase in POMC protein after 10 min UVB irradiation (Figure 1D). Based on the consistent results among multiple assays, 10 min UVB exposure was considered the most effective dose for POMC expression induction in HaCaT keratinocytes and was adopted for all subsequent experiments.

### 3.2 Nicotinic ACh Receptor Activation Affects the UVB‐Induced POMC Expression

**FIGURE 2:**
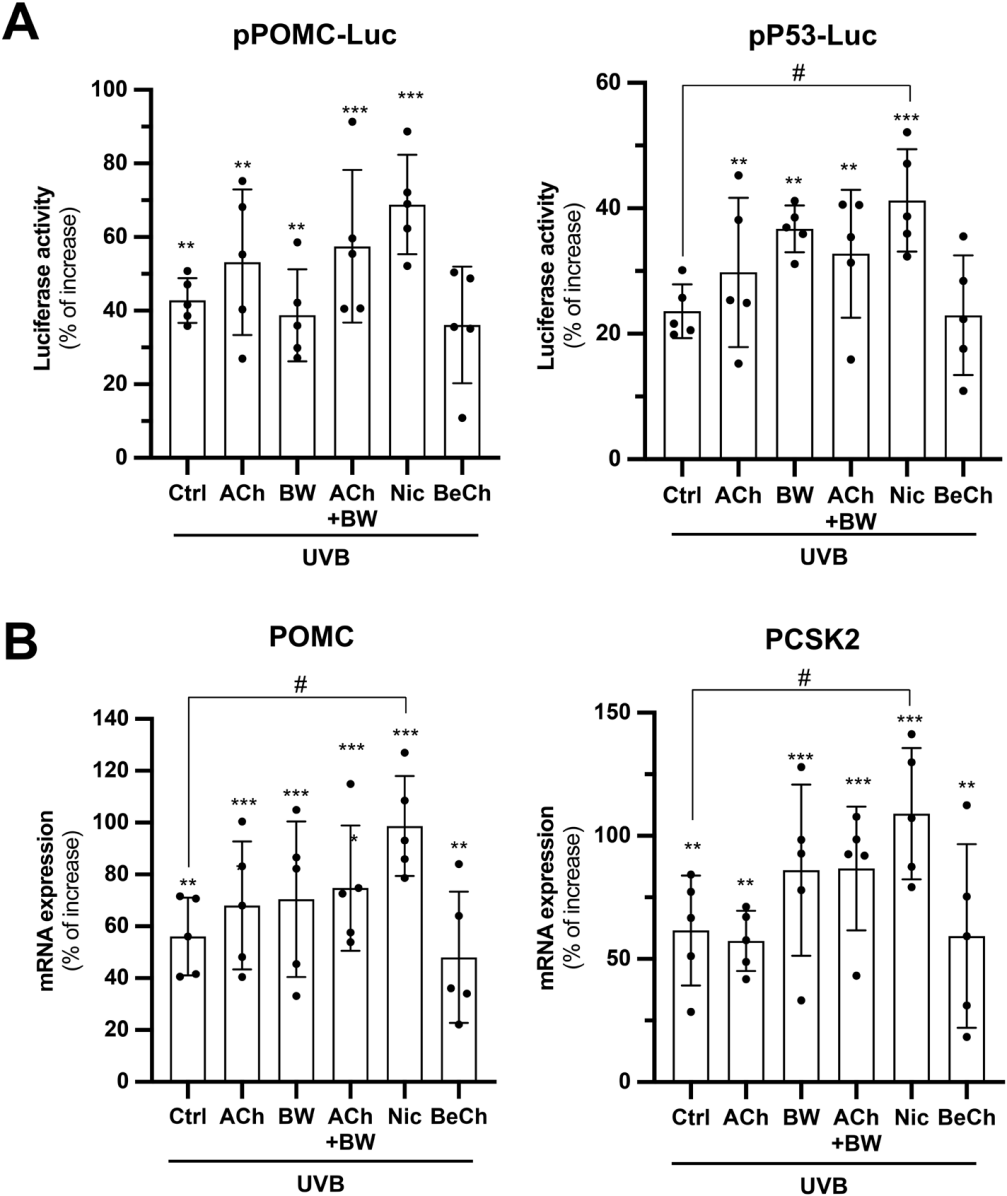
Activation of nAChR potentiates UVB‐induced POMC expression. (A) HaCaT cells were seeded onto 24‐well plates in a density of 1.5 × 10^4^ cells/mL and subjected to transient transfection with pPOMC‐Luc or pP53‐Luc. Collected cell lysates after treatments were subjected to luciferase promoter assay to assess the promoter activity of POMC or p53. (B) The cells were seeded onto 12‐well plates in a density of 1.5 × 10^4^ cells/mL and subjected to drug treatments, followed by RNA extraction and reverse transcription for synthesis of cDNA samples. The cDNA samples were brought to RT‐qPCR for analyzing expression of POMC and PCSK2. The cells were treated with ACh (0.1 mM), BW284c51 (BW; 10 μM), nicotine (Nic; 100 μM) or bethanechol (BeCh; 100 μM) for 3 h as a pretreatment prior to UVB induction (10 min) and were collected 6 h afterwards. Values are expressed as percentage of increase, in Mean ± SD, *n* = 5. Statistical significance was analyzed by one‐way ANOVA, **p < 0.01, ***p < 0.001 versus the blank control; ^#^ *p* < 0.05 versus the UVB control.

To probe the role of the cholinergic system in UVB‐induced POMC expression, ACh receptor‐specific agonists and acetylcholinesterase (AChE) inhibitor were adopted. Agents used included acetylcholine (ACh; pan‐cholinergic agonist), BW284c51 (BW; AChE inhibitor), nicotine (selective nAChR agonist), and bethanechol (BeCh; selective muscarinic acetylcholine receptor (mAChR) agonist). Following pre‐treatment with cholinergic drugs and UVB irradiation, the cells were collected after 6 h incubation. The UVB treatment group served as a positive control. Co‐treating ACh and the AChE inhibitor BW (ACh + BW) further increased the UVB‐induced luciferase activity in cells transfected with either pPOMC‐Luc or p53‐Luc reporters (Figure 2A). By inhibiting AChE, BW prolonged the presence of ACh in the cultures, resulting in a more robust and sustained cholinergic signaling, which implied a role of ACh in regulating POMC expression.

To identify the receptor type mediating the effect, the receptor‐specific agonists were adopted, including nicotine (nAChR agonist) and BeCh (mAChR agonist). Pretreatment with nicotine potentiated the UVB‐induced response more effectively than ACh + BW, that is, increasing POMC and p53 promoter activities by a further 20%–30%. In contrast, the pretreatment with BeCh did not have a significant effect (Figure 2A). The results suggest that rather than mAChRs, nAChRs are the primary regulators of the UVB‐induced POMC expression. In the results of qRT‐PCR, ACh + BW led to an increase in UVB‐induced POMC. Nicotine pretreatment further significantly upregulated the mRNA level of POMC expression, as induced by UVB. Similar to the previous observation in the luciferase promoter assay, BeCh did not show any obvious effect on UVB‐induced POMC mRNA level (Figure 2B).

POMC cleavage leads to α‐MSH production via the activity of pro‐hormone convertase 2 (PC2) [18]. To understand the cholinergic effect on α‐MSH production, the expression level of PC2 was determined by qRT‐PCR. ACh + BW or nicotine pretreatment enhanced UVB‐induced PC2, encoded by the gene PCSK2 (Figure 2B). BeCh did not have an effect. These observations were in line with those made in POMC mRNA expression. Taken together, ACh has a potentiating effect on POMC and α‐MSH productions under the induction of UVB irradiation, and this effect is specifically mediated by nAChRs, rather than mAChRs.

### 3.3 α7 nAChR Regulates the UVB‐Induced POMC Expression

**FIGURE 3:**
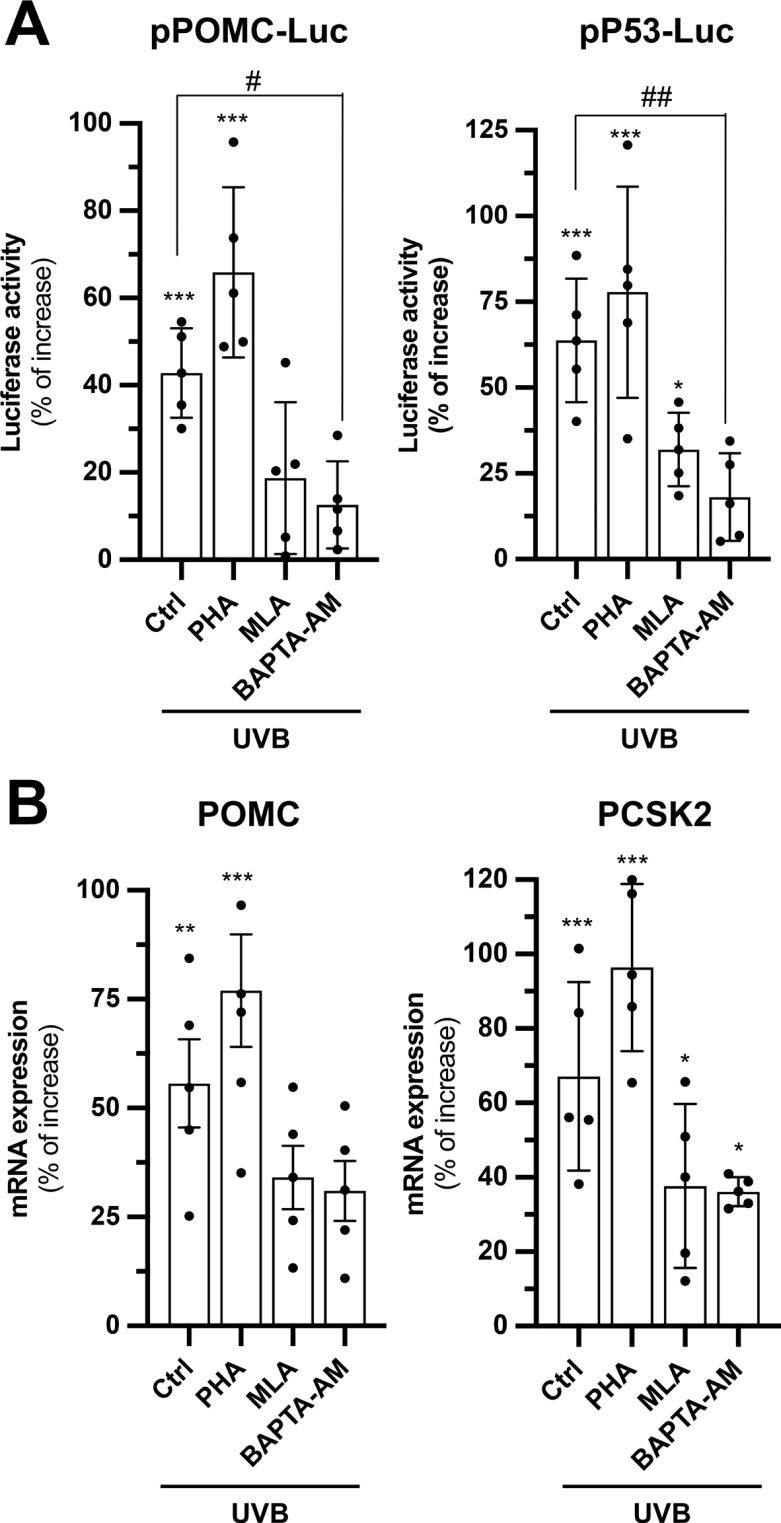
α7 nAChR mediates UVB‐induced POMC expression. (A) HaCaT cells were seeded onto 24‐well plates in a density of 1.5 × 10^4^ cells/mL and subjected to transient transfection with pPOMC‐Luc or pP53‐Luc. Collected cell lysates after treatments were subjected to luciferase promoter assay to assess the promoter activity of POMC or p53. (B) The cells were seeded onto 12‐well plates in a density of 1.5 × 10^4^ cells/mL and subjected to drug treatments. Cells after treatments were collected for RNA extraction and subjected to reverse transcription for cDNA synthesis. The cDNA samples were brought to qRT‐PCR for analyzing expression of POMC and PCSK2. The cells were treated with PHA‐543613 (PHA; 10 μM), methyllycaconitine (MLA; 5 μM) or BAPTA‐AM (20 μM) for 3 h as a pretreatment prior to UVB induction (10 min) and were collected 6 h afterwards. Values are expressed as percentage of increase, in Mean ± SD, *n* = 5. Statistical significance was analyzed by one‐way ANOVA, *p < 0.05, **p < 0.01, ***p < 0.001 versus the blank control; ^#^ *p* < 0.05, ^##^ *p* < 0.01 versus the UVB control.

In epidermal keratinocytes, we reported α7 nAChR has a role in mediating melanosome phagocytosis during pigmentation [10]. The high abundance of α7 nAChR in epidermal keratinocytes suggests that it could be the major subtype of nAChR regulating POMC. Here, PHA‐543613 (PHA; α7 nAChR agonist) and methyllycaconitine (MLA; α7 nAChR antagonist) were deployed in the drug treatment, prior to UVB irradiation for induction of POMC. PHA further increased the POMC and p53 promoter activities, as induced by UVB irradiation. The antagonist MLA and Ca^2+^ chelator BAPTA‐AM showed a suppressing effect on the UVB‐induced activities (Figure 3A). A similar observation was made in the qRT‐PCR results for the mRNA expressions of POMC and PCSK2 (Figure 3B). The expression levels of both transcripts, induced by UVB irradiation, were further enhanced by PHA and reversed by MLA and BAPTA‐AM.

**FIGURE 4:**
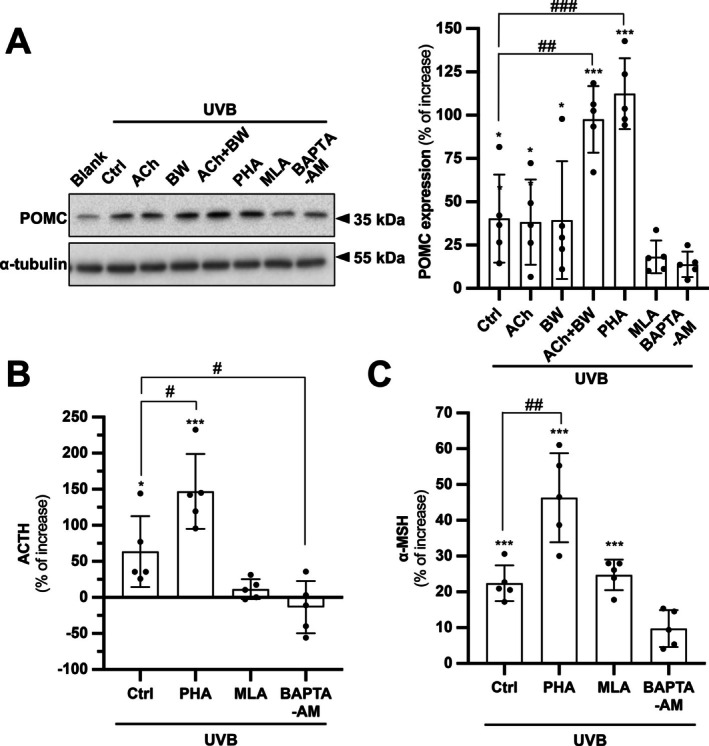
α7 nAChR mediates UVB‐induced POMC protein expression and a‐MSH release. HaCaT cells were seeded onto 12‐well plates in a density of 1.5 × 10^4^ cells/mL and subjected to drug treatments. The cells were treated with ACh (0.1 mM), BW284c51 (BW; 10 μM), PHA‐543613 (PHA; 10 μM), methyllycaconitine (MLA; 5 μM) or BAPTA‐AM (20 μM) for 3 h as a pretreatment prior to UVB induction (10 min) and were collected 6 h afterwards. (A) Samples were subjected to western blotting to assess the expression of POMC at ~35 kDa. α‐tubulin (~55 kDa) was a loading control. Quantification was performed. (B, C) Enzyme‐linked immunosorbent assay (ELISA) was performed to quantify the abundance of corresponding proteins in the conditioned medium. HaCaT cells were seeded onto 12‐well plates in a density of 1.5 × 10^4^ cells/mL and subjected to drug treatments. The conditioned medium was collected for ELISA for the quantification of adrenocorticotropic hormone (ACTH) and α‐melanocyte stimulating hormone (α‐MSH). Values are expressed as percentage of increase, in Mean ± SD, *n* = 5. Statistical significance was analyzed by one‐way ANOVA, *p < 0.05, ***p < 0.001 versus the blank control: ^#^ *p* < 0.05, ^##^ *p* < 0.01, ^###^ *p* < 0.001 versus the UVB control.

These findings at the protein level of POMC were validated by performing Western blot analysis. The exposure to UVB irradiation increased POMC protein expression by approximately 40%. The induction was significantly potentiated by ACh + BW and PHA. In contrast, the increase was suppressed by pretreatment with either MLA or BAPTA‐AM (Figure 4A). The production and release of the POMC‐derived peptides were analyzed by measuring the levels of ACTH (a precursor to α‐MSH) and mature α‐MSH in the conditioned medium. The level of ACTH in conditioned medium was significantly induced by UVB treatment (Figure 4B). PHA treatment had a further significant enhancing effect (~60%) on the production of ACTH induced by UVB. MLA or BAPTA‐AM treatment showed an obvious decrease in ACTH level induced by UVB irradiation (Figure 4B). A similar regulatory pattern was observed in the α‐MSH ELISA result. In parallel, the UVB exposure significantly induced the expression of α‐MSH, which was further significantly potentiated by PHA (Figure 4C).

**FIGURE 5:**
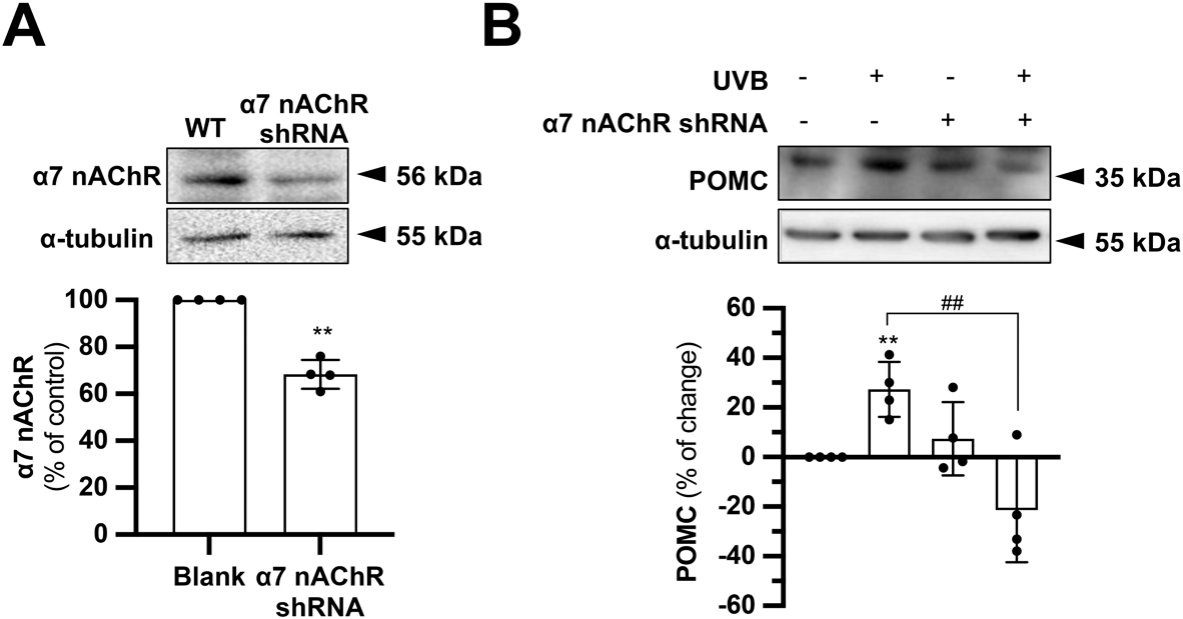
Genetic silencing of α7 nAChR inhibits UVB from inducing POMC in HaCaT keratinocytes. (A) HaCaT cells were seeded onto 6‐well plates in a density of 1.5 × 10^4^ cells/mL and subjected to transiently transfection with α7 nAChR shRNA. The knockdown efficiency was evaluated by western blotting to check the expression level of α7 nAChR (~56 kDa) in shRNA‐transfected cells by comparing to that without transfection (wildtype; WT). Quantification was performed. (B) WT and α7 nAChR shRNA‐transfected HaCaT cells were treated with or without UVB. Western blotting analysis was performed to quantify and compare the expression of POMC (~35 kDa). α‐tubulin (~55 kDa) was the loading control. Values are expressed as percentage of control (or change), in Mean ± SD, *n* = 4. Statistical significance was analyzed by one‐way ANOVA, **p < 0.01 versus the blank control: ^##^ *p* < 0.01 versus the UVB control.

To establish a direct mechanistic link beyond pharmacology, α7 nAChR was genetically silenced using shRNA. Transfection achieved a ~40% reduction in α7 nAChR protein level (Figure 5A). Following shRNA transfection, the protein expression of POMC was assessed as well. While α7 nAChR knockdown alone did not affect the basal POMC expression, it significantly altered the UVB‐induced increase in POMC protein. In α7 nAChR‐depleted cells, the UVB‐induced POMC protein was reduced by ~20%, as compared to the blank control (Figure 5B). This result confirms that α7 nAChR is necessary for the UVB‐induced POMC response. Its presence may be required for maintaining POMC protein stability upon UVB stress in keratinocytes.

**FIGURE 6:**
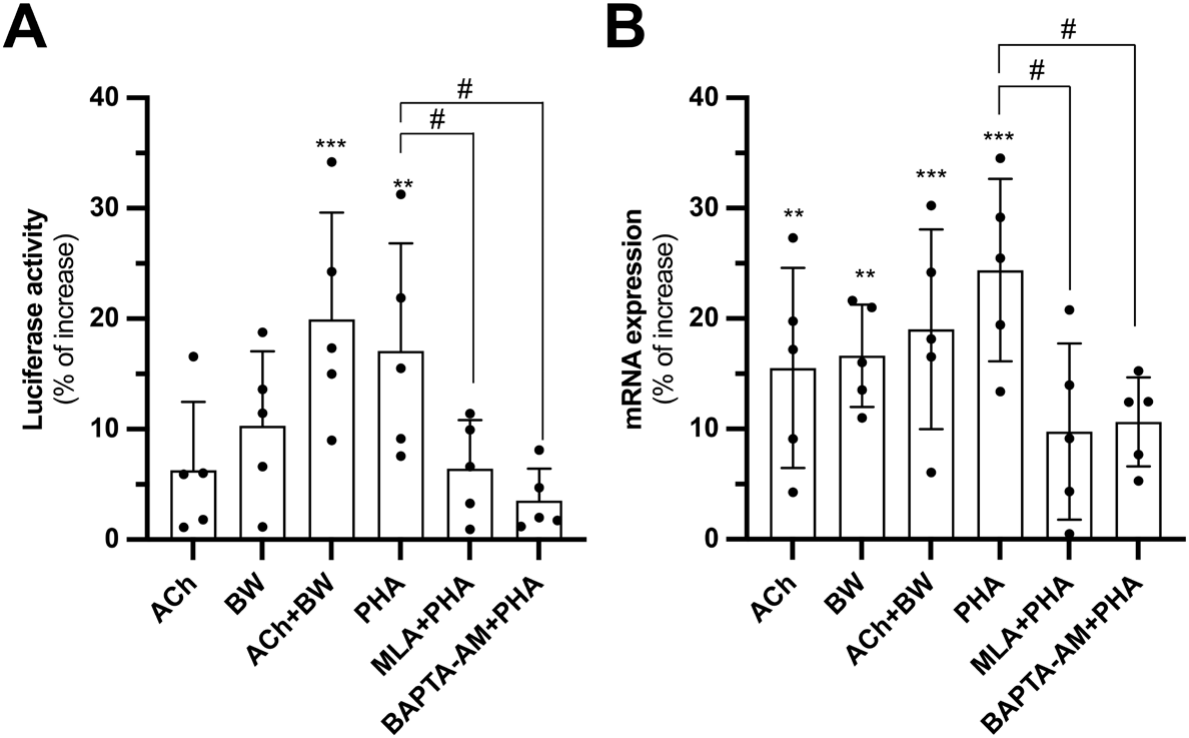
α7 nAChR mediates POMC expression without UVB induction. (A) HaCaT cells were seeded onto 12‐well plates in a density of 1.5 × 10^4^ cells/mL and transiently transfected with pPOMC‐Luc plasmid. The transfected cells were applied with drugs including ACh (0.1 mM), BW284c51 (BW; 10 μM), PHA‐543613 (PHA; 10 μM), methyllycaconitine (MLA; 5 μM) or BAPTA‐AM (20 μM) and collected after 6 h. Luciferase assay was performed to determine the corresponding promoter activity. (B) HaCaT cells were seeded onto 12‐well plates in a density of 1.5 × 10^4^ cells/mL and subjected to drug treatment as described in (A). The cultures were collected for RNA extraction and reverse transcription for cDNA synthesis. The cDNA samples were brought to qRT‐PCR for analysis of POMC expression. Values are expressed as percentage of increase, in Mean ± SD, *n* = 5. Statistical significance was analyzed by one‐way ANOVA, **p < 0.01; ***p < 0.001 versus the blank control; ^#^ *p* < 0.05 versus the PHA treatment group.

Next, we investigated whether cholinergic activation alone, in the absence of UVB irradiation, was able to induce POMC expression. Treatments of ACh + BW and PHA significantly induced POMC promoter activity (Figure 6A) and mRNA expression (Figure 6B). Notably, the inductive effect was much weaker than that induced by UVB irradiation. Both MLA and BAPTA‐AM could alleviate the effect of PHA on POMC expression (Figure 6A,B). Together, the results suggest that the agonists should act through α7 nAChR agonists and the signaling is Ca^2+^‐dependent.

### 3.4 Inhibition of α7 nAChR in Keratinocytes Affects Melanogenesis in Melanoma Cells

**FIGURE 7:**
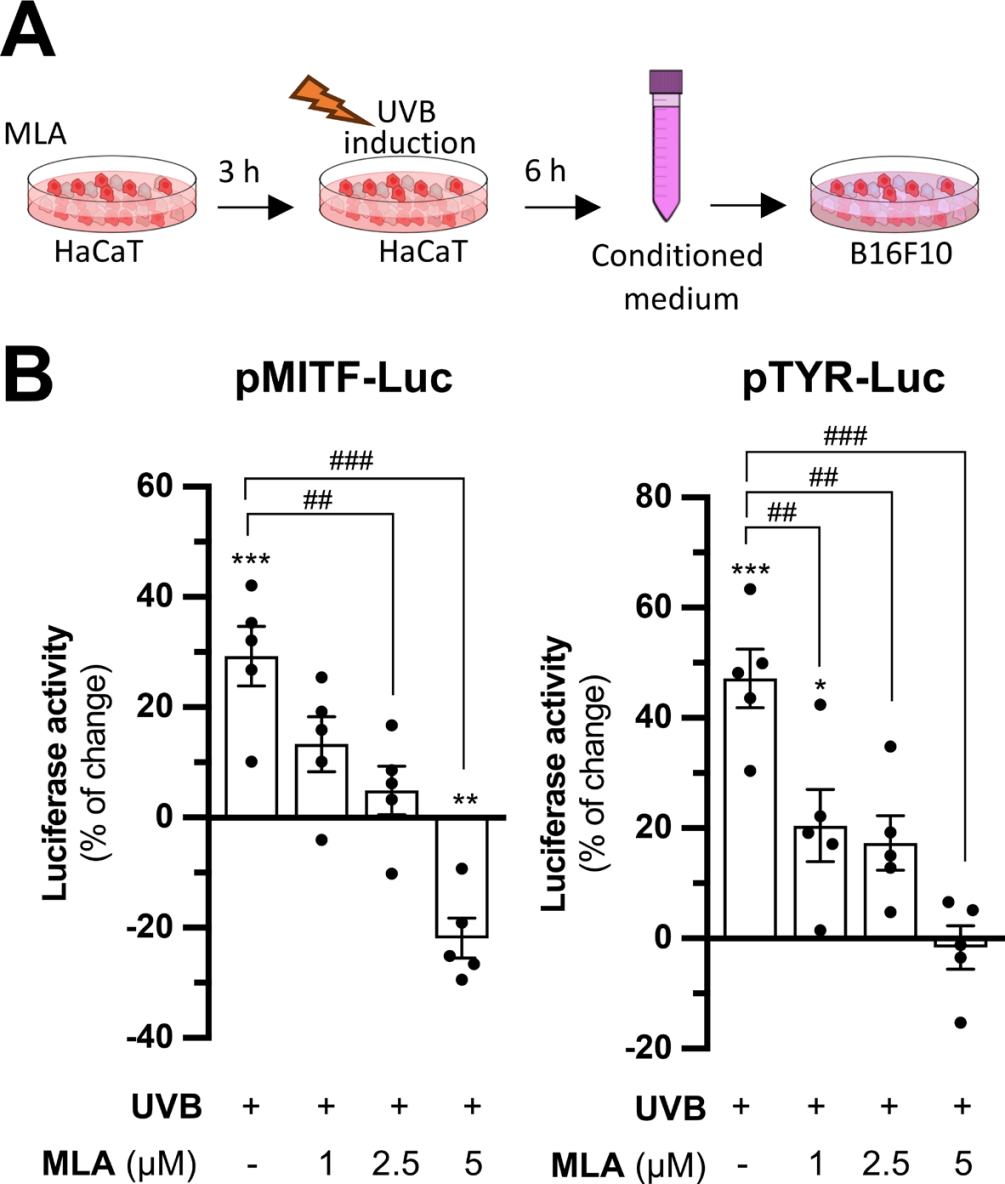
MLA downregulates promoter activity of melanogenic enzymes of B16F10 cells in conditioned medium of HaCaT cells. (A) Flow chart outlining the preparation of conditioned medium from HaCaT keratinocytes to treat B16F10 melanoma cells. HaCaT cells were pretreated with or without methyllycaconitine (MLA) for 3 h and subjected to UVB induction. The conditioned medium was collected after 6 h and treated to B16F10 melanoma cells. The B16F10 cells were collected 24 h afterwards and brought to analysis on melanogenesis. (B) B16F10 cells and HaCaT cells were seeded onto 24‐well plates in a density of 1.0 × 10^5^ cells/mL and 1.5 × 10^5^ cells/mL, respectively. B16F10 cells were transiently transfected with DNA plasmids pMITF‐Luc and pTYR‐Luc. Conditioned medium collected from treated HaCaT cells were collected and applied to transfected B16F10 cells as described in (A). Luciferase promoter assay was performed to determine the promoter activity of MITF and TYR. Values are expressed as percentage of change, in Mean ± SD, *n* = 5. Statistical significance was analyzed by one‐way ANOVA, *p < 0.05, **p < 0.01, ***p < 0.001 versus the blank control; ^##^ *p* < 0.01, ^###^ *p* < 0.001 versus the UVB control.

Having established that α7 nAChR activation is crucial for the UVB‐induced POMC production in keratinocytes, as well as the α‐MSH secretion in conditioned culture medium depending on the cholinergic signaling, we next questioned whether the consequent decrease in α‐MSH secretion could functionally impact melanogenesis. As a natural ligand of the MC1R on melanocytes causing melanogenesis, we hypothesized that the conditioned medium containing α‐MSH could suppress melanogenesis in the recipient B16F10 melanoma cells. HaCaT keratinocytes were pretreated with α7 nAChR antagonist MLA for 3 h prior to UVB irradiation. The conditioned medium was collected 6 h post‐irradiation and applied onto B16F10 melanoma cells (Figure 7A). The treated B16F10 cells after incubation were subsequently subjected to assay the promoter activity, mRNA, and protein expression levels of the melanogenesis‐related target genes, including MITF, TYR, TRP‐1, and DCT (TRP‐2). MITF is the transcription factor mastering the transcription of genes encoding melanogenesis‐related enzymes, TYR, TRP‐1, and TRP‐2.

**FIGURE 8:**
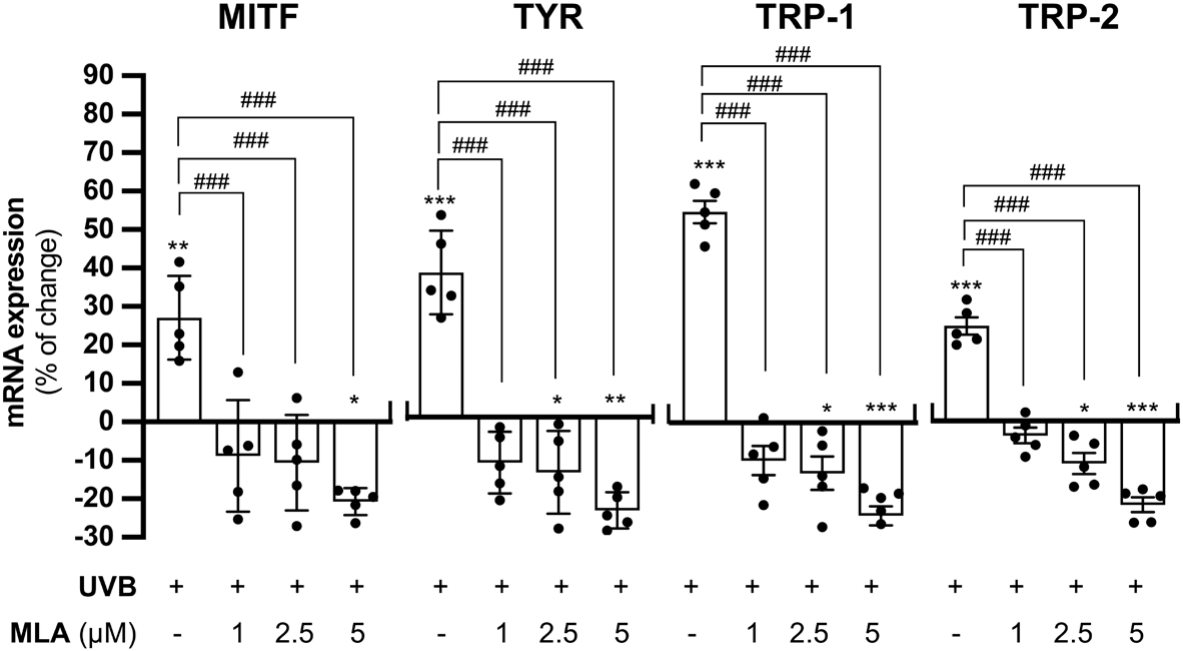
MLA downregulates mRNA expression of melanogenic enzymes of B16F10 cells in conditioned medium of HaCaT cells. B16F10 cells and HaCaT cells were seeded onto 12‐well plates in a density of 1.0 × 10^5^ cells/mL and 1.5 × 10^5^ cells/mL, respectively. Conditioned medium collected from treated HaCaT cells was collected and applied to B16F10 cells as described in Figure 7A. B16F10 cells were collected for RNA extraction and subjected to reverse transcription for cDNA synthesis. The cDNA samples were brought to qRT‐PCR to analyze the expression of MITF, TYR, TRP‐1, and TRP‐2. The expression level of each target transcript was normalized with GAPDH transcript. Values are expressed as percentage of change, in Mean ± SD, *n* = 5. Statistical significance was analyzed by one‐way ANOVA, *p < 0.05, **p < 0.01, ***p < 0.001 versus the blank control; ^###^ *p* < 0.001 versus the UVB control.

The applied conditioned medium from UVB‐exposed HaCaT keratinocytes significantly increased the promoter activities of MITF and TYR in B16F10 cells by approximately 30% and 45%, respectively. This effect was dose‐dependently suppressed when the medium was derived from HaCaT cells treated with MLA and UVB induction. The highest MLA concentration (5 μM) significantly reduced the promoter activity below basal levels (Figure 7B). Moreover, MLA itself had no direct effect on the luciferase activities in the cultured B16F10 cells (Figure S1), supporting the notion that the suppressive effect on promoter activities should be mediated through its action on keratinocytes solely. In the results of RT‐qPCR, the applied conditioned medium of UVB‐induced HaCaT keratinocytes significantly induced the expressions of transcripts encoding MITF, TYR, TRP‐1, and TRP‐2. The transcriptional levels of melanogenic enzymes in the cultures were suppressed after treatment with the conditioned medium collected from HaCaT cells treated with MLA and UVB, and a dose‐dependent pattern in the suppression was observed (Figure 8).

**FIGURE 9:**
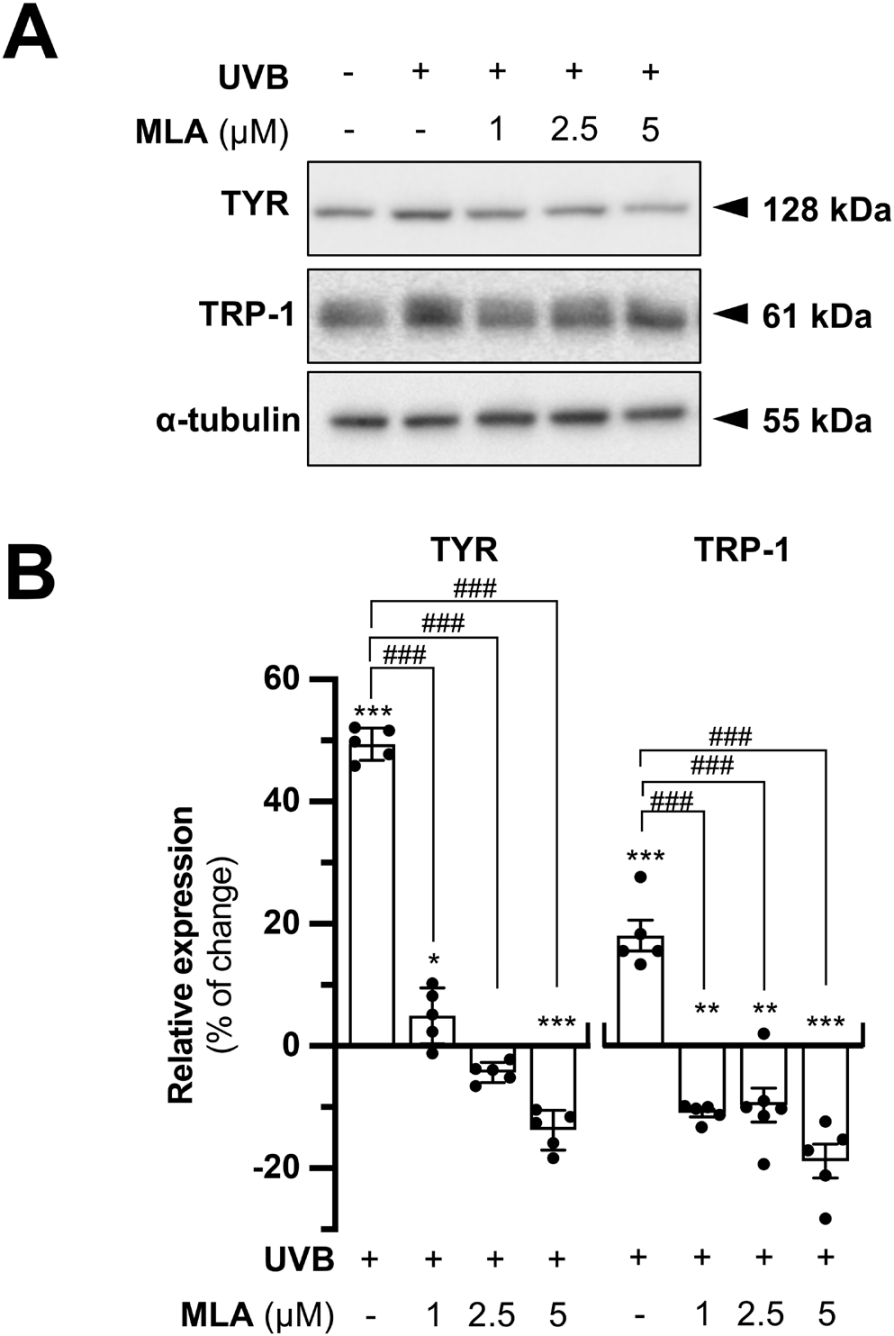
MLA downregulates protein expression of melanogenic enzymes of B16F10 cells in conditioned medium of HaCaT cells. B16F10 cells and HaCaT cells were seeded onto 12‐well plates in a density of 1.0 × 10^5^ cells/mL and 1.5 × 10^5^ cells/mL, respectively. Conditioned medium collected from treated HaCaT cells was collected and applied to B16F10 cells as described in Figure 7A. (A) Expression of proteins TYR (~128 kDa) and TRP‐1 (~61 kDa) was analyzed by performing Western blotting analysis. α‐tubulin was a loading control (~55 kDa). (B) Quantification was performed from (A). Values are expressed as percentage of change, in Mean ± SD, *n* = 5. Statistical significance was analyzed by one‐way ANOVA, **p < 0.01, ***p < 0.001 versus the blank control; ^###^ *p* < 0.001 versus the UVB control.

In addition, the expression of the melanogenic enzymes at the translational level was evaluated by Western blotting analysis. In parallel to the former observations, the collected medium from HaCaT treated with MLA and UVB caused significant suppression of the expressions of TYR and TRP‐1 in dose‐dependent manners (Figure 9A,B). In summary, these results demonstrate that blocking α7 nAChR activation in keratinocytes diminishes the secretion of α‐MSH; this downregulation, consequently, leads to reduced melanin synthesis in melanocytes.

## 4 Discussion

During skin pigmentation, the functional bioactive peptide α‐MSH is derived from enzyme cleavage of POMC and secreted by keratinocytes. Secreted α‐MSH binds to MC1Rs present on melanocytes, activating the signaling pathways linked to melanin synthesis. The produced melanin undergoes transportation to keratinocytes [10, 11], which eventually provides protection against UV irradiation by absorbing and dissipating UV energy, thereby reducing DNA damage [2]. In this study, we further identified a novel role of α7 nAChR in regulating the UVB‐induced POMC production and the subsequent secretion of α‐MSH in epidermal keratinocytes. In addition to the p53‐mediated POMC production under UVB exposure [1], we have shown that cholinergic activation via α7 nAChR has a potentiating effect on this phenomenon. The signaling mechanism is dependent on Ca^2+^ influx. Moreover, the findings have been validated in the functional study on melanogenesis, indicating that inhibiting α7 nAChR activation in keratinocytes could reduce α‐MSH acting on melanocytes, thereby suppressing melanogenesis in the recipient melanocytes. Although the theory of UVB exposure and POMC‐derived α‐MSH production in keratinocytes was established [1], the upstream signaling mechanisms remained unclear. Our study identified the specific receptor, α7 nAChR, as the key modulator in mediating UVB signal with a Ca^2+^‐dependent POMC response, as an expansion in understanding the regulatory mechanisms of this skin pigmentation pathway.

In the hypothalamus, POMC has a more sophisticated role in connecting the hypothalamic, pituitary, and adrenal axis, which is a complex neuroendocrine system modulating the body's stress responses. POMC is primarily synthesized and released by neurons located in the arcuate nucleus of the hypothalamus, which are regulated by input signals from the CNS and peripheral tissues [18]. Studies have demonstrated that the hypothalamic neurons expressing POMC in the arcuate nucleus have cholinergic phenotypes, including the expressions of cholinergic biomarkers, that is, ACh, ChAT, and VAChT [13]. POMC neurons in the arcuate nucleus receive different forms of cholinergic innervation. Nicotine depolarizes the membrane potential and enhances the firing activity of POMC neurons. Both α4β2 and α7 subtypes of nAChRs mediate the responses, as the depolarizing effect of nicotine could be significantly reduced by antagonists targeting the nAChR subtypes [14, 19]. In addition, studies on rats demonstrated that the administration of a high dose of nicotine increased hypothalamic POMC expression, which suggested that nicotine could enhance the synthesis of POMC‐derived peptides in the hypothalamus and other projection areas [20]. In isolated perfused mouse brains, nicotine was shown to enhance the release of POMC‐derived peptides, including α‐MSH, ACTH, and β‐endorphin [21]. As illustrated in this study, a similar regulatory role of α7 nAChR on POMC expression was revealed in the epidermal keratinocytes. Specifically, the receptor's agonist nicotine and PHA significantly potentiated UVB‐induced POMC expression as well as α‐MSH secretion, and the effect could be abolished by the antagonist MLA. Moreover, activating the receptor with the agonist solely could lead to an increase in POMC expression as well, supporting the specific role of α7 nAChR in the activity.

Nicotinic receptors mediate protection against neurotoxicity. For example, the α7 nAChR‐mediated signaling pathways are involved in protection of neurons, macrophages, and microglia [22, 23]. Other than that, studies have reported the role of α7 nAChR in the skin epidermis. Monocytes and macrophages in skin expressing α7 nAChRs could potentially play a role in controlling skin homeostasis [24]. During UV‐induced photoaging or related dermatoses, α7 nAChR agonists have been reported to reduce pro‐inflammatory cytokine production, as well as the reactive oxygen species (ROS)‐mediated collagen degradation in keratinocytes and fibroblasts [24, 25]. In addition to the current knowledge on epidermal α7 nAChR, our current study has supported that this receptor plays a photoprotective role for skin under UV irradiation, which mediates the production and processing of UVB‐induced POMC peptides, that is, α‐MSH, and the subsequent melanogenesis in melanocytes.

The regulation of POMC transcription by pituitary is well recognized; however, the mechanism underlying its activation in non‐pituitary sites remains incompletely elucidated. As a physiologic response to UV irradiation in skin, the activated p53 mainly contributes to the transcriptional regulation of POMC, as well as the secretion of α‐MSH and β‐endorphin [1, 26]. Apart from UVB irradiation, the regulation of POMC expression could be triggered by other pathophysiological responses as well, such as Ca^2+^ influx. As shown here, the potential coupling of Ca^2+^ influx with α7 nAChR activation was illustrated during the regulation of POMC expression in the keratinocytes. The blockage of α7 nAChR by its antagonist, as well as a decreased Ca^2+^ level by Ca^2+^ chelator treatment, led to a downregulation of UVB‐induced POMC transcription. In terms of processing of α‐MSH from POMC, α7 nAChR activation and Ca^2+^ signaling could be involved, as the expression of processing enzyme PC2, encoded by PCSK2, was shown to be regulated in a similar manner. Further investigation is required to elucidate the relationship between Ca^2+^ influx, α7 nAChR regulation, and the signaling activation in mediating UVB‐induced POMC expression in skin keratinocytes.

This current study focused on the specific gene expression changes in epidermal keratinocytes under exposure to UV irradiation. Meanwhile, these findings could be considered in a broader physiological and environmental context. The ambient solar UV irradiation does both benefits and harms to human health, including the synthesis of vitamin D and increased incidence of skin cancer, respectively [27, 28]. Even though how an individual exposes to solar UV irradiation is more likely a personal choice, the environmental factors, such as ozone depletion and climate change, could result in a passive and unintended exposure to a greater amount of UV irradiation [27]. As the primary barrier of the human body, increased UV irradiation imposes more risks in skin homeostasis. Skin‐related carcinomas, such as melanoma and non‐melanoma skin carcinomas (e.g., basal cell carcinoma and squamous cell carcinoma), are commonly known to be associated with UV exposure [29]. Other than these, the non‐cancer UVB‐associated skin conditions are exemplified by immune suppression and photo‐dermatoses (e.g., polymorphic light eruption) [30]. Notably, other climate factors, for example, temperature change due to global warming and ozone depletion, could affect skin condition as well [31]. Here, our results provide more implications given the increasing levels of environmental UV radiation linked to global warming. The mechanisms described here could contribute to understanding the rising incidence of certain skin‐related health issues.

The findings could be linked to implications for understanding certain dermatological aspects of chronic pain conditions as well, such as fibromyalgia syndrome (FMS), which is a chronic pain widespread among muscle, bones, and tendons [32]. Notably, patients with FMS exhibit lower serum levels of choline, suggesting a deficit in the precursor for ACh [33]. Besides, studies have demonstrated UV irradiation, or endogenous opioid release, could have potential in modulating pain in FMS [34, 35]. Thus, the regulatory role of cholinergic signaling in UVB‐induced POMC and POMC‐derived peptide production could lead to further investigation into FMS pathophysiology and the potential topical therapeutic strategies.

## 5 Conclusion

As a conclusion, this study focuses on how cholinergic signaling could regulate the production of POMC, as well as α‐MSH secretion in keratinocytes. Here, the α7 nAChR potentiates POMC expression induced by UVB. The antagonist MLA, Ca^2+^ chelator BAPTA‐AM, or genetic silencing of the receptor suppressed the UVB‐induced POMC expression, supporting the primary role of α7 nAChR in the process. In addition, the conditioned medium collected from MLA and UVB‐treated HaCaT keratinocytes was applied onto B16F10 melanoma cells, as a functional study on melanogenesis. The antagonist could effectively block α‐MSH secretion in HaCaT cells, which subsequently suppressed melanin synthesis in B16F10 cells. Thus, α7 nAChR signaling in keratinocytes plays roles in regulating melanin synthesis through modulation of UVB‐induced POMC production and processing.

## Author Contributions

Conceptualization, M.S.G. and K.W.K.T; methodology, M.S.G., X.W., J.W., and J.G.; investigation, M.S.G., X.W., J.W., and A.D.; writing – original draft, M.S.G., writing – review and editing, M.S.G. and K.W.K.T.; funding acquisition, K.W.K.T., Y.X., and T.T.D.; supervision, K.W.K.T.

## Conflicts of Interest

The authors declare no conflicts of interest.

## Supporting information


**Figure S1:** MLA treatment itself does not affect the promoter activities of MITF and TYR B16F10 cells were seeded onto 12‐well plates in a density of 1.0 × 10^5^ cells/mL. Methyllycaconitine (MLA) was applied to the cells transiently transfected with DNA plasmids pMITF‐Luc and pTYR‐Luc. Luciferase promoter assay was performed to evaluate the promoter activity of MITF and TYR. Values are expressed as folds to basal (*x* Basal), in Mean ± SD, *n* = 3. Statistical significance was analyzed by paired *t*‐test.

## Data Availability

The data that support the findings of this study are available on request from the corresponding author. The data are not publicly available due to privacy or ethical restrictions.
