# Supplementary figures and images for: Regulation of Melanogenesis in Skin Epidermal Keratinocytes via Activation of α7 Nicotinic Acetylcholine Receptor on the Expression of α‐Melanocyte‐Stimulating Hormone
Source: FASEB J. 2025 Oct 12;39(20):e71131. doi: 10.1096/fj.202502535R (PMC12849533; doi:10.1096/fj.202502535R)

8.5 cm

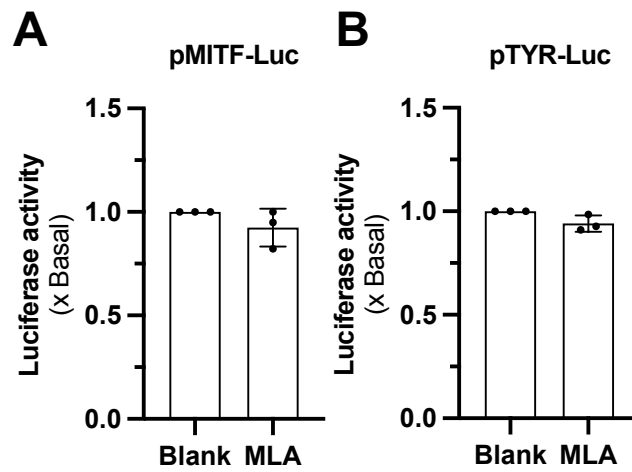

**Supplementary Figure 1**  
**Guo et al., 2025**

Supplement: Supplementary file 1 — Figure S1: MLA treatment itself does not affect the promoter activities of MITF and TYR B16F10 cells were seeded onto 12‐well plates in a density of 1.0 × 10^5^ cells/mL. Methyllycaconitine (MLA) was applied to the cells transiently transfected with DNA plasmids pMITF‐Luc and pTYR‐Luc. Luciferase promoter assay was performed to evaluate the promoter activity of MITF and TYR. Values are expressed as folds to basal (x Basal), in Mean ± SD, n = 3. Statistical significance was analyzed by paired t‐test. [file FSB2-39-e71131-s001.pdf]
